# Relationship between cytokine expression patterns and clinical outcomes: two population‐based birth cohorts

**DOI:** 10.1111/cea.12579

**Published:** 2015-11-19

**Authors:** J. Wu, M. C. F. Prosperi, A. Simpson, E. M. Hollams, P. D. Sly, A. Custovic, P. G. Holt

**Affiliations:** ^1^ Centre for Respiratory Medicine and AllergyInstitute of Inflammation and RepairUniversity of ManchesterUniversity Hospital of South Manchester NHS Foundation TrustManchesterUK; ^2^ Centre for Health InformaticsInstitute of Population HealthUniversity of ManchesterManchesterUK; ^3^ Telethon Institute for Child Health ResearchCentre for Child Health ResearchUniversity of Western AustraliaPerthWAAustralia; ^4^ Queensland Children's Medical Research Institutethe University of QueenslandBrisbaneQldAustralia

**Keywords:** asthma, cytokine pattern, house dust mite allergens, MAAS, Raine Study, sensitization

## Abstract

**Background:**

Models that incorporate patterns of multiple cytokine responses to allergens, rather than individual cytokine production, may better predict sensitization and asthma.

**Objective:**

To characterize the patterns of peripheral blood mononuclear cells’ (PBMCs) cytokine responses to house dust mite (HDM) allergens among children from two population‐based birth cohorts using machine learning techniques.

**Methods:**

PBMCs collected at 8 years of age from the UK Manchester Asthma and Allergy Study (*n* = 268) and at 14 years of age from the Australian Raine Study (*n* = 1374) were cultured with HDM extract (10 μg/ml). Cytokine expression (IL‐13, IL‐5, IFN‐γ, and IL10) was measured in the supernatant. Cytokine patterns were identified using a Gaussian mixture model clustering, and classification stability was assessed by bootstrapping.

**Results:**

A six‐class model indicated complex latent structure of cytokine expression. Based on the characteristics of each class, we designated them as follows: ‘*Nonresponders’* (*n* = 905, 55%); ‘*IL‐10 responders’* (*n* = 49, 3%); ‘*IFN‐*γ *and IL‐13 medium responders’* (*n* = 56, 3.4%); ‘*IL‐13 medium responders’* (*n* = 351, 21.4%); ‘*IL‐5 and IL‐13 medium responders’* (*n* = 77, 4.7%); and ‘*IL‐13 and IL‐5 high responders’* (*n* = 204, 12.4%). ‘IL‐13 and IL‐5 high responders’ were at much higher risk of HDM sensitization and asthma compared to all other classes, with 88% of children assigned to this class being sensitized and 28.5% having asthma.

**Conclusion:**

Using model‐based clustering, we identified several distinct patterns of cytokine response to HDM and observed interplay between cytokine expression level, cytokine patterns (especially IL‐13 and IL‐5), and clinical outcomes. ‘IL‐13 and IL‐5 high responders’ class was strongly associated with HDM sensitization. However, among HDM‐sensitized children, one‐third showed no PBMC response to HDM, and the majority of HDM‐sensitized children did not have asthma or wheeze. Our findings suggest that positive HDM ‘allergy tests’ and asthma are associated with a broad range of immunophenotypes, which may have important implications for the use of cytokine‐targeted treatment approaches.

## Introduction

Sensitization to house dust mite (HDM) allergens is a strong risk factor for childhood asthma, and the titre of HDM‐specific IgE (sIgE) antibodies is a good predictor of asthma presence and persistence 1, 2. However, sensitization is neither necessary nor sufficient for disease expression 3. Among other contributing mechanisms, the expression of asthma may be in part determined by the T‐cell immunity to inhalant allergens 4. In our previous studies characterizing immune responses of peripheral blood mononuclear cells (PBMCs), we have reported that responses to HDM allergens among asthmatic children are heterogeneous, even within the seemingly homogeneous group of children with atopic asthma 5.

Most previous studies evaluated the relationship between individual cytokine responses of PBMCs to mite allergens with clinical outcomes 6, 7. We propose that models that incorporate the patterns of multiple cytokine responses, rather than individual cytokine production, may give a more accurate reflection of the underlying biological processes and better predict the presence of sensitization and wheezing/asthma. Recent developments in machine learning (a data‐driven approach to identify structure within the data using unsupervised learning of latent variables) provide new ways to capture the heterogeneity in patterns of responses to multiple items. Machine learning is increasingly used to disaggregate complex phenotypes in respiratory medicine and allergy 8, 9, 10, 11, 12, 13, 14, 15, where conventional modelling techniques might not uncover the underlying complexity 16.

We hypothesize that there are multiple immunophenotypes of T‐cell immunity to HDM allergens which differ in their relation to mite sensitization and asthma and that machine learning may facilitate better understanding of the patterns of cytokine responses and help identify such immunophenotypes. To address our hypotheses, we characterized the patterns of cytokine responses to HDM allergens using machine learning techniques among children from two large population‐based birth cohorts studied independently in two geographical areas (UK and Australia), amounting to >1600 subjects in whom we measured T‐cell responses.

## Methods

### Study design, setting, and participants

We studied two population‐based birth cohorts, the Manchester Asthma and Allergy Study (MAAS; ISRCTN72673620) 17, 18, 19 and the Western Australia Pregnancy Cohort (Raine) Study 20. Both studies were approved by local research ethics committees. Informed consent was obtained from all parents, and children gave their assent if appropriate.

#### Manchester, UK

MAAS participants were recruited prenatally. Children were followed prospectively and attended review clinic with a clinical assessment and blood collection for cellular studies at eight years of age.

#### Perth, Western Australia

The Raine Study recruited subjects from public antenatal clinics at King Edward Memorial Hospital and nearby private practices. Children were followed prospectively, with a clinical assessment and blood collection at 14 years of age.

### Data sources

Identical validated questionnaires were administered in both cohorts to collect information on parentally reported symptoms, physician‐diagnosed diseases and treatments received 21, 22. We ascertained HDM sensitization by skin prick testing (SPT; Stallergenes, Hauts‐de‐Seine, France) and measurement of HDM‐specific IgE levels using ImmunoCAP^™^ (Phadia AB, Uppsala, Sweden).

### Definition of variables

Clinical outcomes were defined at eight years of age (UK) and at 14 years of age (Australia).

#### Current wheeze

A positive response to the question ‘Has your child had wheezing or whistling in the chest in the last 12 months?’

#### Current asthma

All three of the following: 1) Current wheeze; 2) current use of asthma medication; and 3) physician‐diagnosed asthma ever.

#### Mite sensitization, SPT

Mean HDM SPT wheal diameter ≥3 mm.

#### Mite sensitization, IgE

HDM‐specific IgE > 0.35 kU/L.

### Cellular studies

Cryopreserved PBMCs from Manchester were shipped to Perth for analysis. Although the assays for the two cohorts were not performed simultaneously, the assays were tightly standardized and quality controlled to allow accurate comparison. Coefficients of variation for both cohorts are shown in Table S3. We used identical protocols for *in vitro* studies in both cohorts; the methods are described in detail in our previous studies 5, 20, 23, 24. Briefly, PBMC were cultured for 48 h in AIM‐V medium containing 2‐mercaptoethanol (50 μm) alone (medium control), with phytoheamagglutinin (PHA, 1 μg/ml, positive control), or with HDM (*Dermatophagoides pteronyssinus*) extract (10 μg/ml) which was prepared in‐house, with a single batch used for the whole study (endotoxin level was not quantified). This stimulation time (48 h) was validated in our previous studies 5, 20, 23, 24, 25. IL‐5, IL‐10, IL‐13 and interferon (IFN)‐γ protein in supernatants were assayed using time‐resolved fluorescence as previously described 20. The limit of detection was 10 pg/ml for all cytokines reported in this study. The cytokine response to HDM and PHA was calculated by subtracting the corresponding value from the medium control. Data from PBMC cultures for 16 children were excluded from this analysis – six samples from Raine Study had inadequate viable PBMC numbers for culture, and 10 samples from MAAS study were excluded due to negative response to PHA.

### Statistical methods

Data were modelled separately for each cohort and jointly across the two cohorts.

#### Cytokine class model

Among children with complete data, only those with at least one positive cytokine assay were included in the analysis (*n*_MAAS_ = 119, *n*_RAINE_ = 618). As the distributions of the cytokine values from either cohort were highly skewed, data were transformed (discretized) before clustering. Three types of discretization were performed and tested: binary (positive responder and negative responders); tertile (negative responder, low and high responders); and quartile (negative responder, low, medium, and high responders). Selection of the best discretization method was determined by the overall stability of the cluster structure and the ability of the cluster in exposing most information about the structure of the data set.

Cytokine patterns were identified using a Gaussian mixture model clustering, optimizing parameters by means of the expectation maximization algorithm 26, where the optimal number of clusters was decided by cross‐validation, using the Weka software suite (http://www.cs.waikato.ac.nz/ml/weka/). Model robustness, both in terms of overall clustering structure stability and single cluster support, was analysed by bootstrap analysis (100 times using the R software (http://www.r-project.org/), using the RWeka and e1071 packages). Overall clustering stability was evaluated by calculating the distribution of clustering agreement, measured via the adjusted rand index, a measure of the similarity between two data clusterings, adjusted for the chance grouping of elements 27. The single cluster support was determined by a hard criterion that counted the relative frequency of a cluster being the same (i.e. as determined by the children assigned to it) over all the bootstrap runs.

For each child, we computed a distribution giving the probability of their belonging in each class; for further analysis, we assumed the child belonged to the highest probability class.

#### Association analysis

We then investigated the association between the clinical outcomes and the cytokine levels and the classes we had inferred using the Mann–Whitney *U*‐test and logistic regression analysis. We present the results as the main effect with 95% confidence intervals (CI). Both unadjusted *P*‐values and *P*‐values adjusted using the Bonferroni correction are shown.

## Results

### Participant flow, demographic characteristics and descriptive data

**Figure 1:**
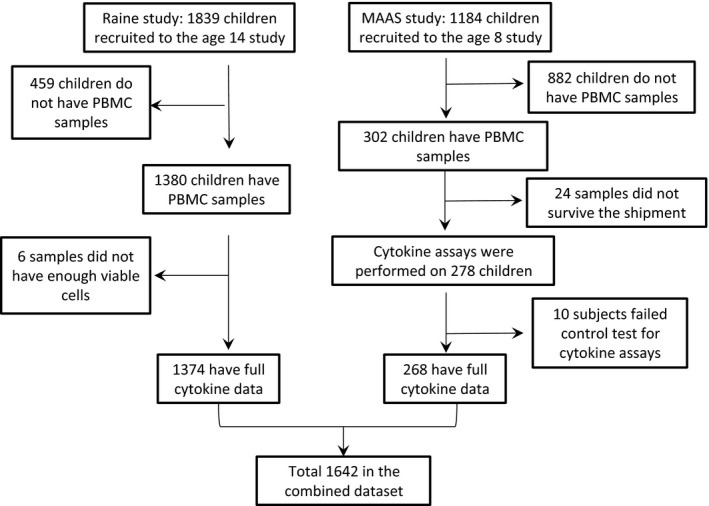
**Consort figure showing participant flow in** **MAAS** **and** **RAINE** **.**

Participant flow is shown in Fig. 1. Of 1184 children born into the MAAS, PBMCs were available for 302; 24 samples had damage to cells as a result of shipping, and a further 10 failed quality control tests (negative PBMC responses to PHA), leaving 268 children with a complete data set. Of these, 58 (21.6%) reported current wheezing, 32 (11.9%) had current asthma, and 58 (21.9%) were sensitized to HDM (Table S1). In the Raine cohort, six samples did not have viable PBMCs for culture and were excluded, and a total of 1374 adolescents had complete data comprising questionnaires and cytokine assays (Fig. 1). Of these, 179 (13.4%) reported current wheezing, 140 (10.5%) had asthma, and 399 (30.1%) were mite sensitized (Table S1). There were no significant differences in demographic characteristics or any of the outcomes between the children who were included and excluded from this analysis (Table S2).

#### HDM‐induced cytokine expression

The proportion of children with positive cytokine responses and the cytokine levels in two cohorts are shown in Table S3. IL‐5 and IL‐13 levels were correlated within each cohort (*R* ^2^ > 0.35), with no significant correlation for other cytokines. The relationship between cytokine levels and clinical outcomes in the two cohorts is shown in Table 1. Expression of IL‐5 and IL‐13 was significantly higher among children with asthma, wheeze or mite sensitization compared to those without these features (*P* < 0.001). For IFN‐γ and IL‐10, levels were significantly higher among mite‐sensitized than nonsensitized children, but there were no difference between children with or without wheeze or asthma.

**Table 1:** Cytokine expression level from merged cohort by clinical outcomes

MAAS & RAINE	Current asthma			Current wheeze			Mite sensitization (SPT)			Mite sensitization (IgE)		
	Mean rank	*P* value	Adjusted *P* value	Mean rank	*P* value	Adjusted *P* value	Mean rank	*P* value	Adjusted *P* value	Mean rank	*P* value	Adjusted *P* value
IL‐5												
No	773.68	<0.001	<0.001	771.80	<0.001	<0.001	693.54	<0.001	<0.001	682.74	<0.001	<0.001
Yes	1008.81			962.09			1042.41			1014.81		
IL‐13												
No	772.73	<0.001	<0.001	770.92	<0.001	<0.001	684.29	<0.001	<0.001	663.70	<0.001	<0.001
Yes	1016.64			967.14			1065.28			1047.05		
IL‐10												
No	795.49	0.25	1.000	802.79	0.45	1.000	766.01	<0.001	<0.001	762.43	<0.001	<0.001
Yes	828.07			783.97			863.21			879.80		
IFN‐γ												
No	791.97	0.02	1.000	800.35	0.93	1.000	756.39	<0.001	<0.001	755.27	<0.001	<0.001
Yes	857.27			797.98			886.98			891.94		

*P* values were calculated by Mann–Whitney test.

Adjusted *P* value were calculated by Bonferroni correction.

### Cytokine clustering and characterization of the cytokine classes

#### The choice of the optimum data transformation (discretization)

We performed initial analyses in the two cohorts separately. We carried out the analyses using three different transformations of the data set (binary, tertiles, quartiles; Tables S4, S5). The robustness of the binary solution was low (ARI score from bootstrapping; ARI_MAAS_: 0.2, ARI_RAINE_: 0.46). In contrast, clusters generated from tertile‐ and quartile‐discretized data had similar structure and high stability (ARI_MAAS_: 0.48–0.55, ARI_RAINE_: 0.66–0.68). Tertile discretization showed higher robustness of the clusters than that from the quartile discretization, and we observed similar patterns across the two cohorts (Tables S4, S5). We therefore discretized the combined data sets into tertiles before performing joint clustering.

#### Cytokine classes identified

**Figure 2:**
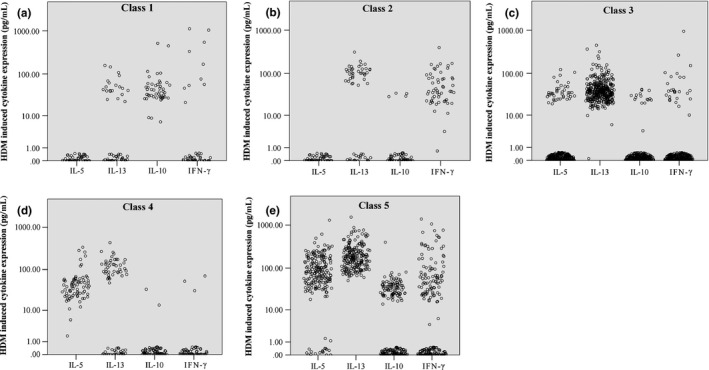
Cytokine expression pattern of individual cytokine class (a–e). Scatter plot showing cytokine expression pattern within each cytokine classes.

**Figure 3:**
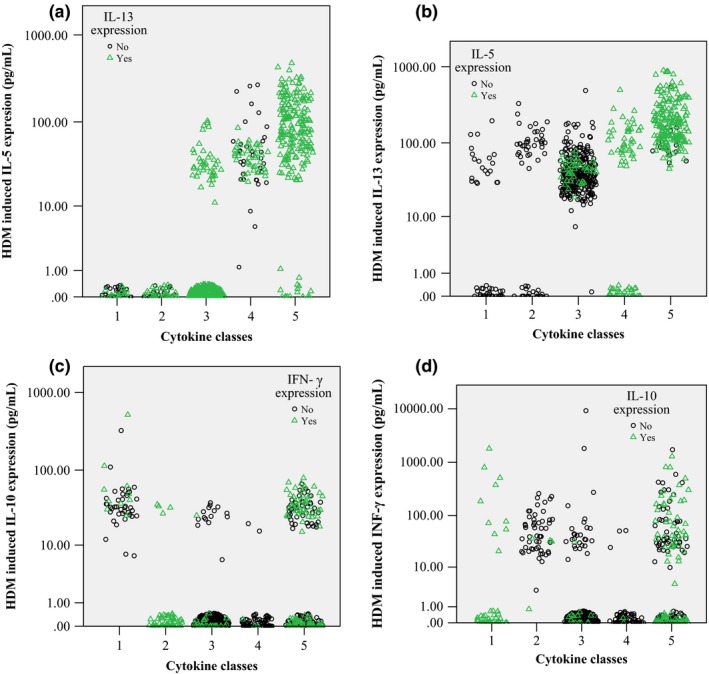
Expression level of individual cytokine in each of the cytokine classes. Scatter plot showing IL5 (a), IL13 (b), IL10 (c), and IFN‐γ (d) expression level between cytokine classes.

Children negative for all cytokines were *a priori* assigned to Class 0 (*‘Nonresponder’*,* n* = 905, 55%). We then applied the Gaussian mixture model clustering to all data from children with a positive response for at least one cytokine (*n* = 737). The optimal model that best described the combined data sets was a five‐class model (Table S6, ARI = 0.85). The distribution of children across the five cytokine classes was comparable between MAAS and Raine (Table S6). Based on our interpretation of their characteristics (Figs 2 and 3), we assigned the classes as:

*Class 1, ‘IL‐10 responder’ (n = 49, 3%)*. All children in this class produced IL‐10 (Fig. 2a), with 20 also producing IL‐13 and ten producing IFN‐γ; none of the children in this class expressed IL‐5 (Figs 3a and b).
*Class 2, ‘IFN‐*γ *and IL‐13 medium responder’ (n = 56, 3.4%)*. All children in this class produced IFN‐γ (Fig. 2b), and the majority (*n* = 37) also produced IL‐13, but not IL‐5 (Figs 3 and b).
*Class 3, ‘IL‐13 medium responder’ (n = 351, 21.4%)*. All children produced IL‐13, but only few produced other three cytokines (Figs 2c and 3a–d).
*Class 4, ‘IL‐5 and IL‐13 medium responder’ (n = 77, 4.7%)*. The majority of children assigned to this class co‐expressed IL‐13 and IL‐5 (*n* = 43), with only few (*n* = 5) producing IFN‐γ or IL‐10 (Fig. 2d). There were no IFN‐γ and IL‐10 co‐expressers (Figs 3c and d).
*Class 5, ‘IL‐13 and IL‐5 high responder’ (n = 204, 12.4%)*. The majority of children in this class (*n* = 188) co‐expressed IL‐13 and IL‐5, and 141 also produced IFN‐γ or IL‐10 (Fig. 2e). Children in this class produced the highest levels of IL‐13 and IL‐5 (Figs 3a and b).

### Cytokine classes and clinical outcomes

The association between cytokine classes and clinical outcomes is presented in Table 2. Although some children in Class 1 and Class 2 produced IL‐13, the clinical outcomes in these two classes were not significantly different from the ‘Nonresponders’ in Class 0.

**Table 2:** Wheeze, asthma, and mite sensitization among children in different cytokine classes

Classes (*n*)	Current wheeze			Current asthma			Mite sensitisation (SPT)			Mite sensitisation (IgE)		
	*n* (% positive)	OR (95% CI)	*P* value (Adjusted)	*n* (% positive)	OR (95% CI)	*P* value (Adjusted)	*n* (% positive)	OR (95% CI)	*P* value (Adjusted)	*n* (% positive)	OR (95% CI)	*P* value (Adjusted)
0 (905)	883 (9.9)			857 (6.8)			875 (16.2)			890 (22.4)		
1 (49)	48 (16.7)	1.64 (0.62–4.31)	0.31 (1)	47 (10.6)	1.82 (0.82–4.03)	0.13 (1)	49 (14.3)	0.89 (0.43–1.81)	0.74 (1)	49 (20.4)	0.86 (0.37–1.95)	0.71 (1)
2 (56)	53 (9.4)	1.46 (0.56–3.83)	0.43 (1)	52 (9.6)	0.95 (0.36–2.45)	0.92 (1)	52 (21.2)	1.27 (0.68–2.34)	0.44 (1)	56 (26.8)	1.38 (0.69–2.75)	0.35 (1)
3 (351)	344 (15.1)	1.73 (1.12–2.68)	0.01 (0.53)	336 (11.2)	1.62 (1.12–2.35)	0.009 (0.38)	340 (32.4)	2.93 (2.24–3.81)	<0.001 (<0.001)	343 (45.8)	2.46 (1.84–3.29)	<0.001 (<0.001)
4 (77)	74 (21.6)	3.9 (2.07–7.35)	<0.001 (0.001)	69 (22.1)	2.52 (1.39–4.58)	0.002 (0.09)	72 (48.6)	4.77 (2.94–7.73)	<0.001 (<0.001)	76 (57.9)	4.88 (2.97–8.01)	<0.001 (<0.001)
5 (204)	197 (35.0)	5.49 (3.62–8.33)	<0.001 (<0.001)	192 (28.5)	4.93 (3.41–7.11)	<0.001 (<0.001)	199 (76.4)	25.02 (15.87–39.45)	<0.001 (<0.001)	197 (87.8)	16.69 (11.49–24.24)	<0.001 (<0.001)

Logistic regression was used for calculating the odds ratio.

‘Class 0’ was used as the reference group.

By investigating classes 3, 4, and 5, we observed interplay between cytokine expression level, cytokine patterns (especially IL‐13 and IL‐5), and clinical outcomes. Children in Class 3 (moderate IL‐13 production in response to HDM allergen) were at a modest risk of mite sensitization compared to Class 0 (OR 2.93, 95% CI 2.24–3.81, *P* < 0.001), but did not differ significantly from Class 0 in relation to wheeze or asthma (*P* = 0.53 and 0.38, respectively).

The expression of IL‐13 seemed to be the prerequisite for IL‐5 expression, as children who produced IL‐5, but not IL‐13, were rare (part of Class 4, <2% of study population). The majority of children who produced IL‐5 (in classes 4 and 5) had accompanying high‐level expression of IL‐13 (relative to Class 3), and high levels of IL‐5 were associated with high prevalence of asthma, wheeze and mite sensitization (Table 2, classes 4 and 5).

**Figure 4:**
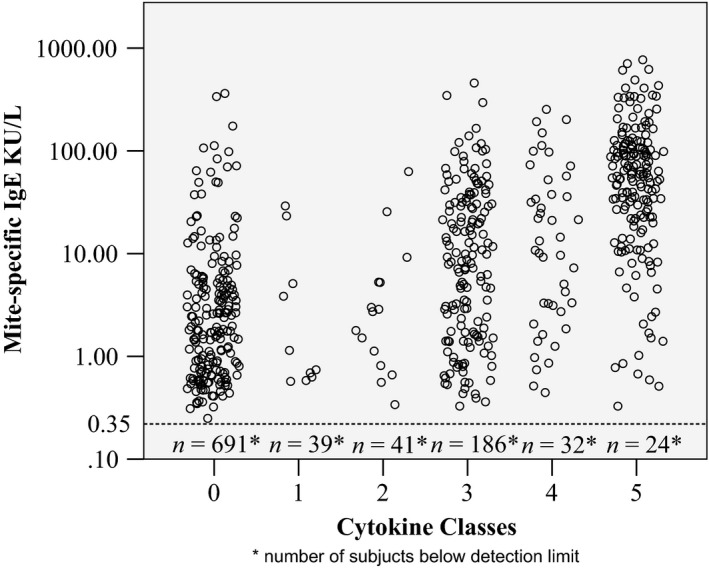
Mite‐specific IgE level by cytokine classes. Scatter plot showing mite‐specific IgE level of children >0.35 kU/L between cytokine classes in a log scale. Cut‐off level of 0.35 kU/L is indicated by the dotted line. The numbers below the dotted line are the numbers of children with mite‐specific IgE ≤ 0.35 kU/L in each cytokine class.

Children in Class 5 were at a considerably higher risk of mite sensitization (OR 25.02, 95% CI 15.87–39.45), with 88% of children assigned to this class being sensitized (IgE). The absolute levels of mite‐specific IgE were significantly higher in Class 5 compared to other four classes, both in the whole group and among sensitized children only (Fig. 4, *P* < 0.001). However, despite the fact that most children in Class 5 were mite sensitized and that this class was strongly associated with asthma (OR 4.93, 95% CI 3.41–7.11) and wheeze (OR 5.49, 95% 3.62–8.33), the majority of children in this class did not have asthma or wheeze (71.5% and 65%, respectively, Fig. S1). At the population level, the majority of children who were sensitized to mite, or had asthma or wheezing, came from Class 0 (Table S7).

## Discussion

### Key results

To our knowledge, this is the first study to analyse patterns of PBMC cytokine responses to HDM allergens *in vitro* by applying unsupervised machine learning methodologies to jointly model the data, rather than investigate associations for each cytokine separately. We identified five distinct cytokine response classes to the four cytokines we measured (IL‐5, IL‐10, IL‐13, and IFN‐γ) in children from two population‐based birth cohorts. These classes differed significantly in their association with HDM‐specific sensitization, asthma, and wheeze. We observed different associations for wheeze/asthma compared to HDM sensitization.

### Limitations

We acknowledge that this is likely a simplified model of the immune responses to HDM allergens by PBMC, with only four cytokines taken into account. Other cytokines such as IL‐4, IL‐9, IL‐25, and IL‐33, thymic stromal lymphopoietin, as well as other cytokine‐producing cells such as type 2 innate lymphoid cells, eosinophils, airway epithelial cells, and mast cells (within tissue) also play important roles in mediating HDM‐induced immune response 20, 28, 29, 30.

We have not taken into account the inter‐individual differences in PBMC composition. PBMCs are a mixed cell population of lymphocytes, monocytes, and dendritic cells. Although a mixed cell population is a more accurate representation of what happens *in vivo*, knowing the composition of the mixed cell population (e.g. by using cell sorting) might provide further insight into biological mechanisms. Likewise, we have chosen to focus on a single early (48 h) time point post‐allergen stimulation, as opposed to later time points at which cell proliferation and cytokine secretion peaks, in order to minimize the ‘bystander’ effects of allergen‐specific memory cell‐derived cytokines on other cell populations present within the mixed PBMC cultures. It is possible that additional information of interest could be obtained by inclusion of additional time points, but this is not within the scope of this study.

Our two data sets were obtained from different ages and geographical areas. To minimize ‘batch effect’, all samples were shipped to Australia for analysis. To further reduce heterogeneity, we analysed data from the two cohorts separately and determined the optimal transformation of the data before merging the cohorts for the final clustering. The distribution of cytokine classes generated from merged data was almost identical between the two cohorts, suggesting that clustering was not driven by any one cohort (Table S6).

### Interpretation

Our data are suggestive of an interplay between cytokine expression level, cytokine patterns (in particular IL‐13 and IL‐5), and clinical outcomes.

#### Sensitization and immune responses to HDM

Almost all children in Class 5 were sensitized to HDM, suggesting that high expression of both IL‐13 and IL‐5 by HDM‐stimulated PBMCs is one of the major pathological features of sensitization. However, only 35% of these children had current wheeze and 28.5% were asthmatic, implying that HDM sensitization is a risk factor, but not necessarily *per se* on the causal pathway in the pathogenesis of asthma and wheeze. Among HDM‐sensitized children, one‐third showed no response to HDM in the cell culture experiments, a further third were in Class 5, and the rest were spread across the remaining four classes. These findings indicate that a positive ‘allergy test’ can be associated with a broad range of immunophenotypes.

#### Asthma and immune response to HDM

Children in Class 5 (all of whom produced high levels of IL‐13, and almost all produced high levels of IL‐5, with some production of IL‐10 and IFN‐γ) had a fivefold increase in the risk of asthma. After adjustment for multiple testing, none of the other classes were significantly associated with asthma. Class 4 was significantly associated with wheeze, and we observed a nonsignificant trend for the association with asthma. However, among children with asthma at a community level, the majority (one‐third) came from the Class 0 (negative for all four cytokines), almost one‐third were in the Class 5, and the remaining asthmatics were spread across the other four classes (Table S7).

#### Interpretation of classes and potential mechanisms

The large proportion of nonresponders was unlikely the result of suboptimal culture condition, as culture conditions were stringently maintained across each cohort, and all analysed subjects had at least one positive cytokine expression under PHA stimulation. In addition, the proportion of nonresponders was very similar between the two cohorts, despite age and geographic differences, implying that HDM‐stimulated PBMC responses are reproducible. It is likely that the frequency of circulating HDM‐specific T memory cells was too low in a subset of HDM‐sensitized individuals to elicit a detectable cytokine response to HDM in PBMC culture; given the high level of quality control maintained to ensure consistency across all PBMC preparations, cultures and cytokine measurements within each cohort, this lack of detected HDM response can be viewed as an indicator of immune phenotype diversity. Furthermore, though rigorous, our investigation of immune responses to HDM exploration was not exhaustive, and there are pathways outside of those we have studied that contribute to HDM allergy.

In the current study, we observed highly coordinated expressions of IL‐13 and IL‐5 in classes 4 and 5 (with higher levels seen in Class 5). *IL‐5* and *IL‐13* genes are located in a close proximity in the Th2 locus, which also contains the *IL‐4* and the constitutively expressed *Rad50* gene 31. The entire locus is ~140 kb long and is similar between humans and mice 32. In mouse T cells, remodelling of the chromatin structure within this region occurs when naive T helper cells differentiate into mature Th2 cells, an event not observed during Th1 differentiation 33. It has been proposed that this mechanism is important for the coordinated expression of these two cytokines, also resulting in potentiation of the expression of the two cytokines 34, 35. We speculate that the co‐expressors may have remodelled chromatin structure within this region.

In PBMC culture, the expression of IL‐5, IL‐10, IL‐13, and IFN‐γ is mediated by different cell types. Whereas IL‐5 and IL13 are expressed mainly by the Th2 cells 36, IL‐10 is mainly produced by regulatory T cells 37 and potentially monocytes 38 and IFN‐γ by natural killer and natural killer T cells, as well as CD4 Th1 and CD8 cytotoxic T cells 39. Children exhibiting different cytokine profiles from *in vitro* cultured PBMC not only reflect the possible change of cellular function, but may also indicate altered cellular composition in the immune system.

Among the ‘IL‐13 and IL‐5 high expressors’ (Class 5), the levels of IL‐10 and IFN‐γ were raised in many of the children. In our previous studies in the RAINE cohort in which each cytokine was analysed individually within HDM‐sensitized adolescents, higher levels of IL‐10 and IFN‐γ were associated with an increased risk of asthma 20. In the current analysis, higher IL‐10 or IFN‐γ was only associated with asthma in the context of high IL‐5 and IL‐13 productions; children who produced IL‐10 but no IL‐5 (Class 1), or IFN‐γ but no IL‐5 (Class 2) were not at increased risk of asthma. Mechanistically, little is known about what may underlie this phenomenon. However, in mouse T cells, multiple physical interactions between the ‘IFN‐γ locus’ and ‘Th2 locus’ have been observed. Deletion of one of the interaction sites (*RHS7*) significantly delays IFN‐γ expression and reduces IL‐5 expression 40. Whether the co‐expression of IL‐13, IL‐5 and IFN‐γ was a result of inter‐chromosomal interaction requires further investigation.

The strong association between the co‐expression of IL‐13 and IL‐5 with asthma or wheeze reflects the functionality of the two cytokines, which has been demonstrated in mouse aeroallergen challenge models, where airway remodelling, airway inflammation, and AHR were dramatically reduced after the knockout of either IL‐13 or IL‐5 41, 42. In addition, as IL‐13 is one of the main inducers for IgE production 43, the high expression levels of IL‐13 in our Class 5 may account for the high HDM sIgE titres within this class. However, it is noteworthy that 28 subjects were not sensitized to HDM, despite the high expression of both IL‐13 and IL‐5. Focusing on these children in the future study might give further insights into the mechanisms that naturally counterbalance the biological effect of IL‐13 and IL‐5.

Around a quarter of children were ‘IL‐13 single expressors’ (Class 3 and the majority of Class 2). In contrast, ‘IL‐5 single expressors’ were rare (~2% of the study population). This implies that most subjects express IL‐5 under the ‘coordinated expression model’. In contrast, low levels of IL‐13 expression without activating IL‐5 were common, suggesting two different expression mechanisms between ‘IL‐5/IL‐13 co‐expressors’ and ‘IL‐13 single expressors’. The IL‐13 and IFN‐γ coexpressors (Class 2) showed much lower risks for HDM sensitization compared to Class 3, where the majority did not express IFN‐γ. In the sensitization model, mice receiving recombinant IFN‐γ or intravenous IFN‐γ gene showed reduced antigen‐induced eosinophillic airway infiltration and inhibited development of AHR, suggesting a possible protective role for IFN‐γ 44, 45.

Children in Class1 (‘IL‐10 expressors’) had a relatively low risk of HDM sensitization, asthma, and wheeze. This is biologically plausible and may reflect the immune‐modulatory role of IL‐10. Of note, although the nonresponders (Class 0) had low risk of HDM sensitization as a group, at a population level ~one‐third of HDM‐sensitized children came from this subgroup; we observed similar findings for wheeze/asthma (Table S7).

## Conclusion

Using model‐based clustering approach, we identified several distinct patterns of PBMC cytokine response to HDM allergens. We observed interplay between cytokine levels, the expression patterns, and the manifestation of clinical outcomes. High expression of both IL‐13 and IL‐5 by HDM‐stimulated PBMC was highly associated with HDM sensitization (ascertained by SPT or mite IgE). However, a third of HDM‐sensitized children were in the nonresponder group, suggesting the presence of other mechanisms of HDM sensitization. Even in Class 5, the majority of children did not have current asthma or wheeze, despite the high prevalence of HDM sensitization. The heterogeneity of cytokine patterns shown in this study may have important implications for the efficacy of asthma and allergy treatment approaches that block action of single cytokines.

## Funding source

The Australian study has been supported principally by a series of grants from the National Health and Medical Research Council of Australia. The UK study was funded by UK Medical Research Council (MRC) Grants G0601361 and MR/K002449/1, JP Moulton Charitable Foundation and National Institute for Health Research Clinical Research Facility at University Hospital of South Manchester NHS Foundation Trust.

## Conflict of interests

JKW received a travel grant (GBP400) from BSACI for attending BSACI annual meeting at 2013. AS reports grants from Medical Research Council and Moulton Charitable Foundation, during the conduct of the study; personal fees from GlaxoSmithKline and Chiesi, outside of the submitted work. AC reports grants from Medical Research Council and Moulton Charitable Foundation, during the conduct of the study; personal fees from Circassia, GlaxoSmithKline, Thermo Fisher Scientific, Novartis, ALK, Airsonett, and MSD outside of the submitted work. Remaining authors have no conflict of interest to report.

## Supporting information


**Table S1.** Percentage of children with positive clinical outcomes at follow up age in MAAS and RAINE cohortS.
**Table S2.** Demographic characteristics of children included and excluded in the current study.
**Table S3.** Descriptive analysis of the levels of four cytokines in two cohorts.
**Table S4.** Cytokine classes generated from binary, tertile or quartile discretized data for the MAAS cohort.
**Table S5.** Cytokine classes generated from binary, tertile or quartile discretized data for the RAINE cohort.
**Table S6.** Characterization of the cytokine classes and the proportion of subjects across the classes within the merged data set and in individual cohorts.
**Table S7.** Proportion of positive clinical outcomes across cytokine classes.
**Figure S1.** Correlations between cytokines from MAAS cohort, Raine cohort and merged data, all negative responders were replaced with a value zero.
