# Supplementary material for: Relationship between cytokine expression patterns and clinical outcomes: two population‐based birth cohorts
Source: Clin Exp Allergy. 2015 Nov 19;45(12):1801–11. doi: 10.1111/cea.12579 (PMC4950290; doi:10.1111/cea.12579)

**Relationship Between Cytokine Expression Patterns and Clinical Outcomes: Two Population-based Birth Cohorts**

**ONLINE DATA SUPPLEMENT**

Table S1 Percentage of children with positive clinical outcomes at follow up age in MAAS and RAINE cohortS.

|  | MAAS | | RAINE | |
| --- | --- | --- | --- | --- |
|  | n | n (%) with outcome | n | n (%) with outcome |
| Gender (Male) | 268 | 151 (56.3) | 711 | 368 (51.7) |
| Current asthma | 268 | 32 (11.9) | 1329 | 140 (10.5) |
| Current wheeze | 268 | 58 (21.6) | 1331 | 179 (13.4) |
| Mite sensitization (SPT) | 264 | 58 (21.9) | 1323 | 399 (30.1) |
| Mite sensitization (IgE) | 237 | 59 (24.9) | 1374 | 539 (39.2) |

Table S2. Demographic characteristics of children included and excluded in the current study

|  | **Included** | **Excluded** | **p-value** |
| --- | --- | --- | --- |
| **MAAS; age 8 years follow up** | **n=268** | **n=916** |  |
| Male; n (%) | 151(56.3) | 497(54.2) | 0.55 |
| Current wheeze; n (%) | 58(21.6) | 127(16.8) | 0.08 |
| Current asthma; n (%) | 32 (11.9) | 70 (9.2) | 0.2 |
| Sensitized to dust mite (SPT); n (%) | 58 (22.0) | 132 (20.0) | 0.48 |
| Sensitized to dust mite (IgE); n (%) | 59 (24.9) | 87(25.3) | 0.91 |
| Maternal asthma ever; n (%) | 54 (20.1) | 181(19.7) | 0.89 |
| Paternal asthma ever; n (%) | 43 (16.0) | 120 (13.1) | 0.22 |
| Maternal hay-fever; n (%) | 69 (25.8) | 243 (26.5) | 0.82 |
| Paternal hay fever; n (%) | 67(25.0) | 213 (23.3) | 0.57 |
| Older sibling with asthma; n (%) | 36 (13.5) | 100 (11.0) | 0.27 |
| Mother sensitized to dust mite (SPT); n (%) | 112 (44.3) | 392 (43.9) | 0.92 |
| Father sensitized to dust mite (SPT); n (%) | 123 (49.0) | 403 (45.5) | 0.32 |
| **RAINE: age 14 years follow-up** | **n=1374** | **n=465** |  |
| Male; n (%) | 711(51.7) | 232 (49.9) | 0.48 |
| Current asthma; n (%) | 140 (10.5) | 56 (12.1) | 0.35 |
| Current wheeze; n (%) | 179 (13.4) | 74 (15.9) | 0.18 |
| Sensitized to dust mite (SPT); n (%) | 399 (30.2) | 55(29.7) | 0.91 |
| Maternal asthma ever (at recruitment); n (%) | 184 (13.7) | 62 (13.6) | 0.93 |
| Paternal asthma ever (at recruitment); n (%) | 106 (7.7) | 46 (9.9) | 0.14 |
| Older sibling, asthma ever (at recruitment); n (%) | 111 (8.1) | 32 (6.9) | 0.41 |
| Maternal hayfever ever (at follow-up); n (%) | 361 (27.8) | 115 (25.6) | 0.36 |
| Paternal hayfever ever (at follow-up); n (%) | 206 (18.7) | 82 (20.7) | 0.38 |

Table S3. Descriptive analysis of the levels of four cytokines in two cohorts

|  |  | **IL-5** | **IL13** | **IL10** | **IFN-γ** |
| --- | --- | --- | --- | --- | --- |
| **MAAS** | Positive responder; n (%) | 71 (26.5) | 92 (34.3) | 18 (6.7) | 34 (12.7) |
|  | Mean | 116.19 | 130.30 | 52.38 | 476.44 |
|  | Median | 82.00 | 62.50 | 36.50 | 121.00 |
|  | Minimum | 1.00 | 20.00 | 7.00 | 14.00 |
|  | Maximum | 475.00 | 771.00 | 375.00 | 6092.00 |
| **RAINE** | Positive responder; n (%) | 235 (17.1) | 563 (41) | 143 (10.4) | 160 (11.6) |
|  | Mean | 77.46 | 102.92 | 37.41 | 82.99 |
|  | Median | 52.64 | 62.83 | 29.99 | 41.86 |
|  | Minimum | 1.08 | 0.37 | 5.13 | 1.51 |
|  | Maximum | 328.76 | 944.66 | 365.40 | 1118.56 |
| Inter-assay % Coefficients of Variability for both cohorts | | 6.9 | 7.6 | 8.3 | 11.3 |
| Intra-assay % Coefficients of Variability for both cohorts | | 6.6 | 4.8 | 3.3 | 5.8 |

Table S4 Cytokine classes generated from binary, tertile or quartile discretized data for the MAAS cohort

| MAAS | Number of clusters | Brief description of each subgroup | n | Current wheeze | Current asthma | Mite sensitization (SPT) | Mite sensitization (IgE) | ARI |
| --- | --- | --- | --- | --- | --- | --- | --- | --- |
|  | class 0 | Non-responder | 149 | 14.1% | 5.4% | 6.1% | 10.4% |  |
| Binary | class 1 | No distinguishing feature | 97 | 34.0% | 21.6% | 44.8% | 45.7% | 0.72 |
|  | class 2 | No distinguishable feature | 22 | 18.2% | 13.6% | 28.6% | 36.4% | 0.28 |
|  | Overall ARI |  |  |  |  |  |  | 0.20 |
| Tertile | class 1 | IL5/IL13 low responder | 73 | 20.5% | 13.7% | 22.5% | 24.6% | 0.67 |
|  | class 2 | IL5/Il13 high responder | 46 | 47.8% | 30.4% | 71.7% | 76.3% | 0.79 |
|  | Overall ARI |  |  |  |  |  |  | 0.55 |
| Quartile | class 1 | IL13 single responder | 52 | 17.3% | 11.5% | 18.0% | 19.6% | 0.72 |
|  | class 2 | IL5/IL13 medium responder | 44 | 38.6% | 22.7% | 50.0% | 48.7% | 0.71 |
|  | class 3 | IL5/Il13 high responder | 23 | 47.8% | 34.8% | 78.3% | 94.4% | 0.53 |
|  | Overall ARI |  |  |  |  |  |  | 0.48 |

Table S5 Cytokine classes generated from binary, tertile or quartile discretized data for the RAINE cohort

| RAINE | Number of clusters | Brief description of each subgroup | n | Current wheeze | Current asthma | Mite sensitization (SPT) | Mite sensitization (IgE) | ARI |
| --- | --- | --- | --- | --- | --- | --- | --- | --- |
|  | class 0 | Non responder | 756 | 9.0% | 6.7% | 18.3% | 24.5% |  |
| Binary | class 1 | No distinguishable feature | 446 | 18.5% | 14.8% | 45.3% | 59.0% | 0.71 |
|  | class 2 | No distinguishable feature | 172 | 20.0% | 16.4% | 43.1% | 52.9% | 0.46 |
|  | Overall ARI |  |  |  |  |  |  | 0.46 |
| Tertile | class 1 | IL10 responder | 41 | 17.5% | 10.0% | 17.1% | 43.9% | 0.46 |
|  | class 2 | IL13 single responder | 305 | 14.1% | 10.4% | 32.3% | 68.5% | 0.93 |
|  | class 3 | IFN-γ/IL13 low-medium responder | 54 | 9.8% | 9.8% | 25.0% | 63.0% | 0.52 |
|  | class 4 | IL5 /IL13 medium responder | 117 | 27.0% | 23.4% | 70.9% | 88.9% | 0.49 |
|  | class 5 | IL13/ IL5 medium-high responder | 101 | 29.9% | 25.8% | 74.5% | 93.1% | 0.39 |
|  | Overall ARI |  |  |  |  |  |  | 0.68 |
| Quartile | class 1 | IL10 responder | 35 | 17.6% | 11.8% | 20.0% | 22.9% | 0.36 |
|  | class 2 | IFN-γ / IL13 low-medium responder | 261 | 13.7% | 10.2% | 29.0% | 44.4% | 0.8 |
|  | class 3 | IL13 single responder | 131 | 16.4% | 11.7% | 54.8% | 64.1% | 0.45 |
|  | class 4 | IL5 /IL13 medium responder | 55 | 28.1% | 25.8% | 61.8% | 73.5% | 0.41 |
|  | class 5 | IL13/IL5 medium-high responder | 136 | 29.4% | 25.5% | 69.8% | 83.6% | 0.48 |
|  | Overall ARI |  |  |  |  |  |  | 0.66 |

Table S6 Characterization of the cytokine classes and the proportion of subjects across the classes within the merged data set and in individual cohorts.

| **Classes** | | **Total cases** | | | **Cases from MAAS** | | **Cases from RAINE** | |
| --- | --- | --- | --- | --- | --- | --- | --- | --- |
|  |  | **N** | **%** | **ARI** | **n** | **%** | **n** | **%** |
| 0 | Non-responder | 905 | 55.1% |  | 149 | 55.6% | 756 | 55.0% |
| 1 | IL-10 responder | 49 | 3.0% | 0.68 | 8 | 3.0% | 41 | 3.0% |
| 2 | IFN-γ and IL-13 medium responder | 56 | 3.4% | 0.63 | 7 | 2.6% | 49 | 3.6% |
| 3 | IL-13 single responder | 351 | 21.4% | 0.97 | 46 | 17.2% | 305 | 22.2% |
| 4 | IL-5 and IL-13 medium responder | 77 | 4.7% | 0.42 | 21 | 7.8% | 56 | 4.1% |
| 5 | IL-13 and IL-5 high responder | 204 | 12.4% | 0.58 | 37 | 13.8% | 167 | 12.2% |
|  | Overall ARI |  |  | 0.85 |  |  |  |  |
| Total |  | 1642 | 100.0% |  | 268 | 100.0% | 1374 | 100.0% |

Table S7 Proportion of positive clinical outcomes across cytokine classes

| **Class** | **Total**  **(n and %)** | | **Current wheezer**  **(n=237)** | **Current asthmatic**  **(n=172)** | **Mite sensitized (SPT)**  **(n=457)** | **Mite sensitized (IgE)**  **(n=589)** |
| --- | --- | --- | --- | --- | --- | --- |
| 0 | 905 | 55.1% | 36.7% | 33.1% | 31.1% | 33.3% |
| 1 | 49 | 3.0% | 3.4% | 2.9% | 1.5% | 1.7% |
| 2 | 56 | 3.4% | 2.1% | 2.9% | 2.4% | 2.5% |
| 3 | 351 | 21.4% | 21.9% | 21.5% | 24.1% | 26.3% |
| 4 | 77 | 4.7% | 6.8% | 8.7% | 7.7% | 7.4% |
| 5 | 204 | 12.4% | 29.1% | 30.8% | 33.3% | 28.9% |
| total | 1642 | 100% | 100% | 100% | 100% | 100% |

Figure S1. Correlations between cytokines from MAAS cohort, Raine cohort and merged data, all negative responders were replaced with a value zero.


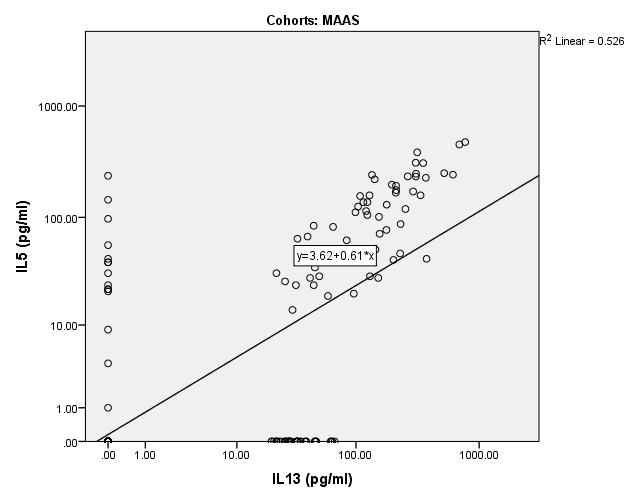


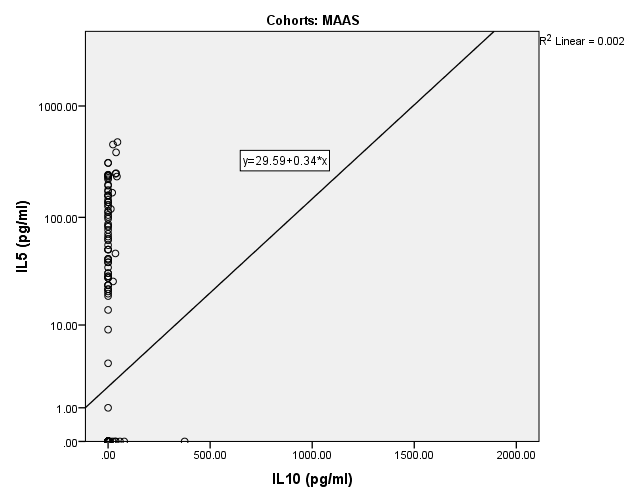


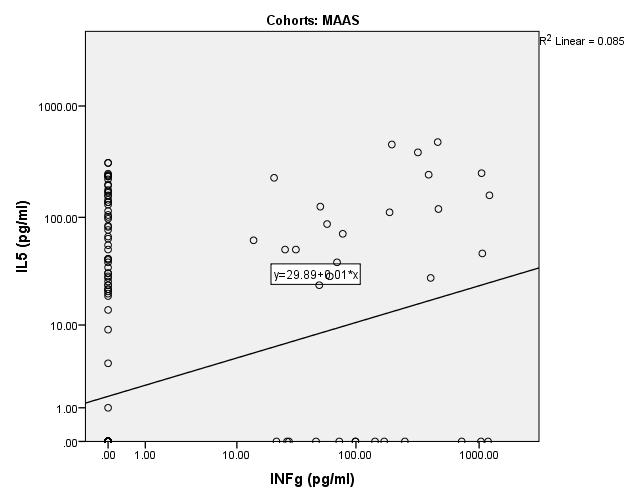


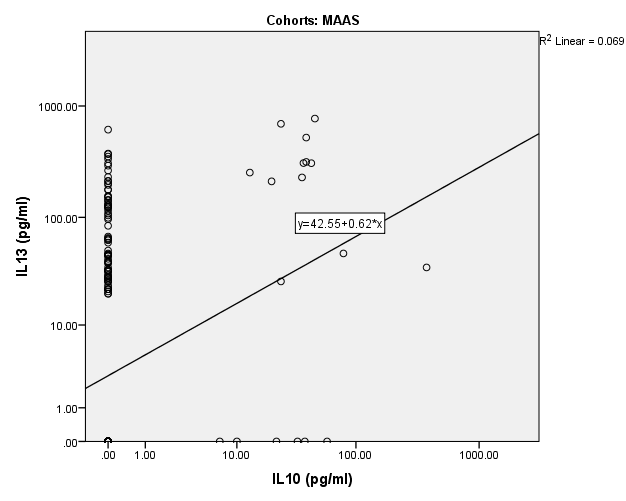


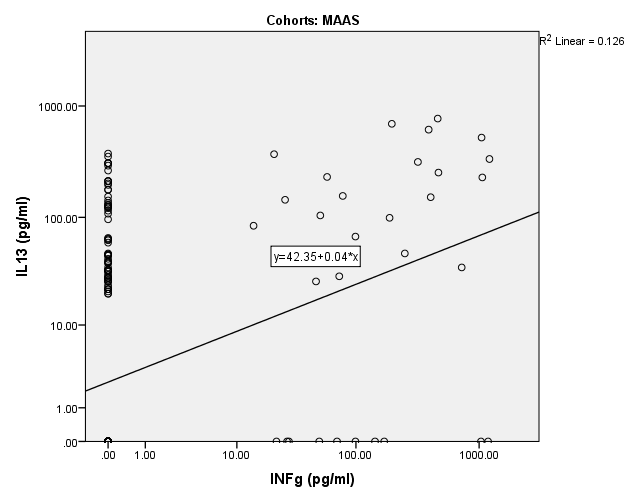


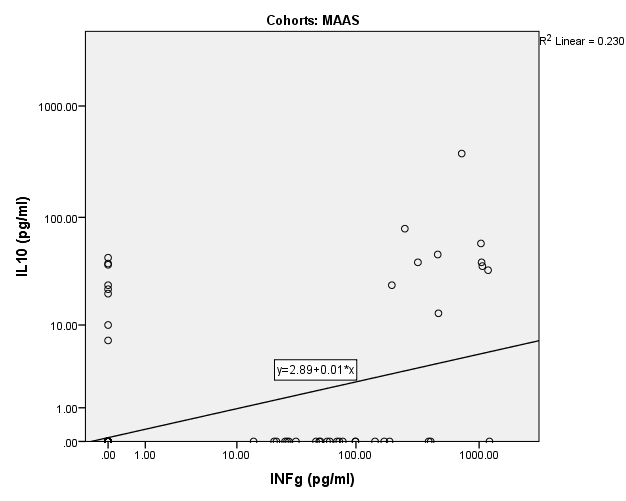


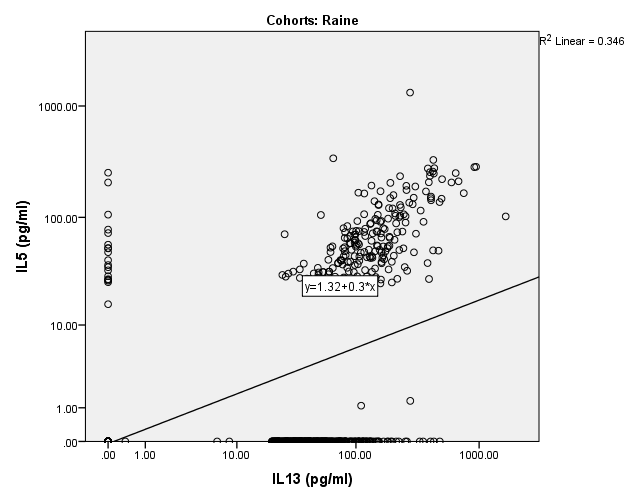


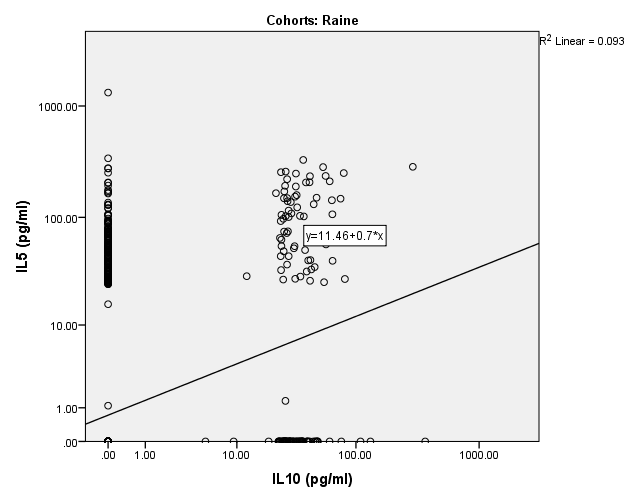


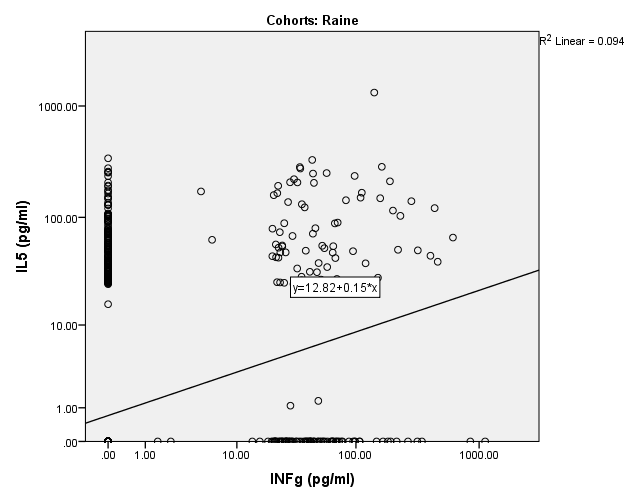


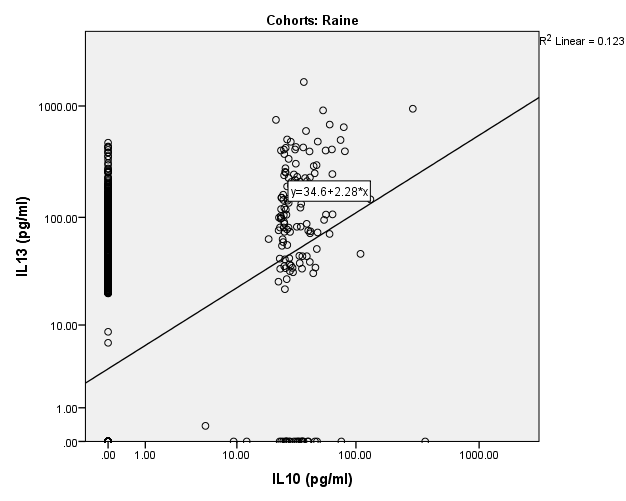


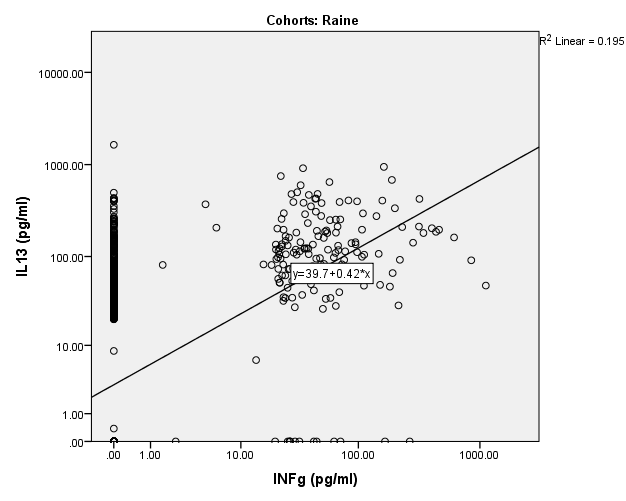


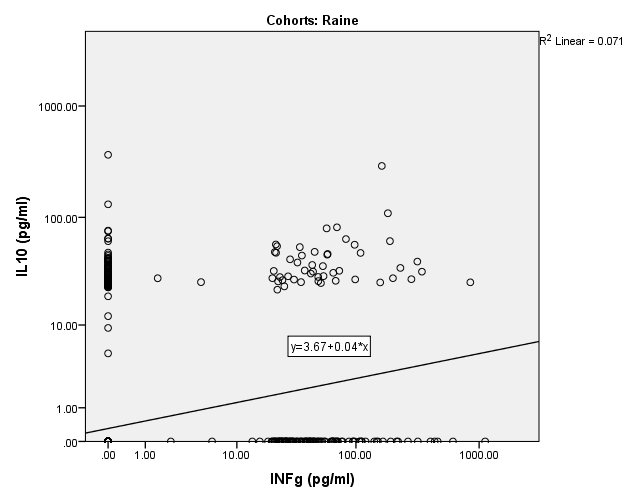


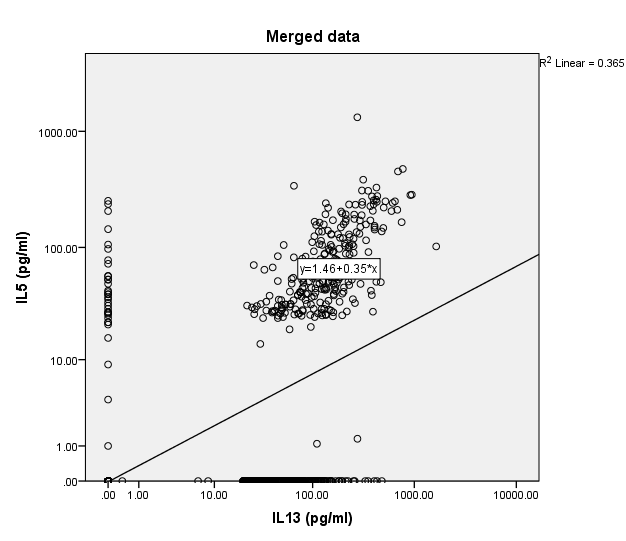


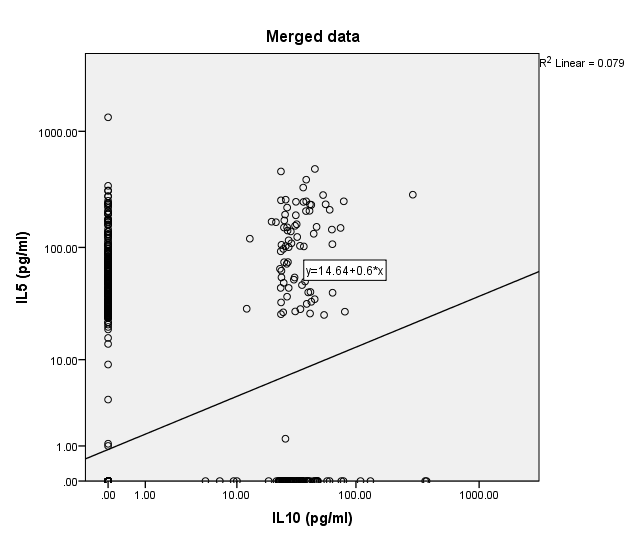


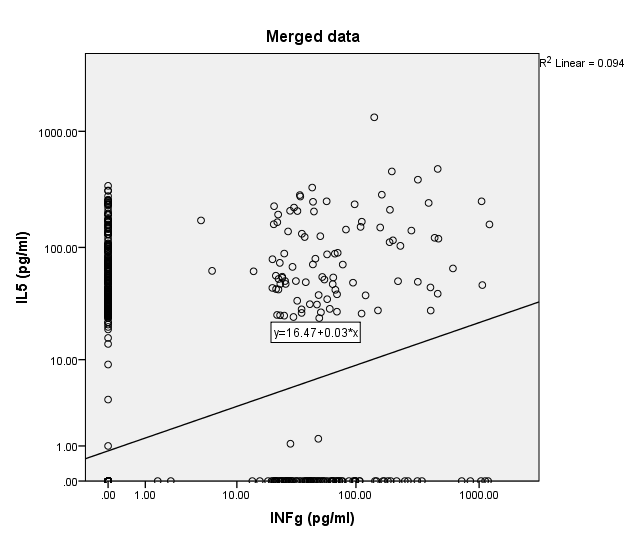


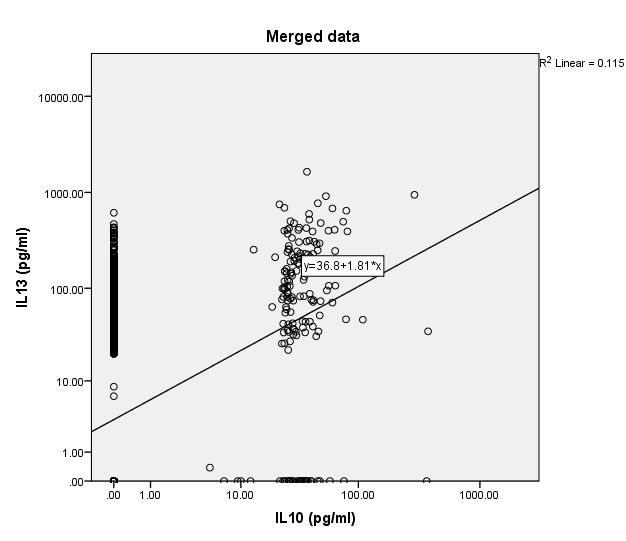


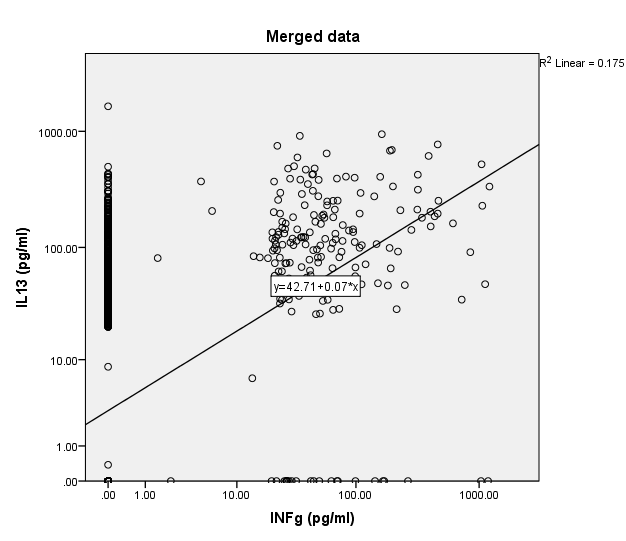


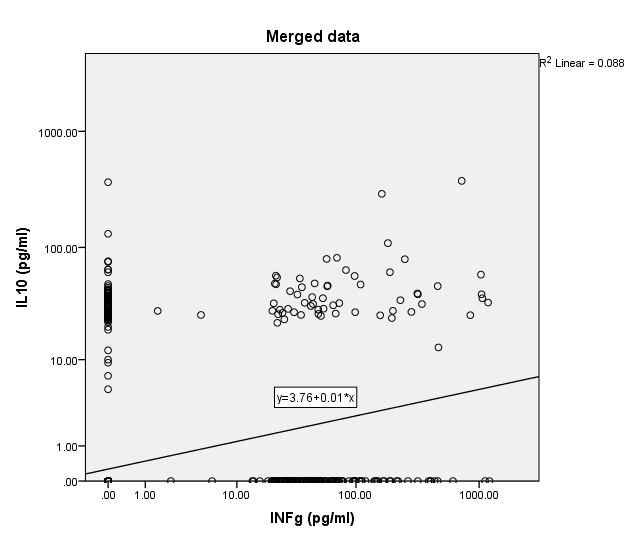

Supplement: Supplementary file 1 — Table S1. Percentage of children with positive clinical outcomes at follow up age in MAAS and RAINE cohortS. Table S2. Demographic characteristics of children included and excluded in the current study. Table S3. Descriptive analysis of the levels of four cytokines in two cohorts. Table S4. Cytokine classes generated from binary, tertile or quartile discretized data for the MAAS cohort. Table S5. Cytokine classes generated from binary, tertile or quartile discretized data for the RAINE cohort. Table S6. Characterization of the cytokine classes and the proportion of subjects across the classes within the merged data set and in individual cohorts. Table S7. Proportion of positive clinical outcomes across cytokine classes. Figure S1. Correlations between cytokines from MAAS cohort, Raine cohort and merged data, all negative responders were replaced with a value zero. [file CEA-45-1801-s001.docx]
